# The PIT: SToPP Trial—A Feasibility Randomised Controlled Trial of Home-Based Physiotherapy for People with Parkinson's Disease Using Video-Based Measures to Preserve Assessor Blinding

**DOI:** 10.1155/2012/360231

**Published:** 2011-10-20

**Authors:** Emma Stack, Helen Roberts, Ann Ashburn

**Affiliations:** ^1^Faculty of Medicine, Academic Geriatric Medicine, University of Southampton, Southampton SO17 1BJ, UK; ^2^Southampton General Hospital, Mailpoint 886, Tremona Road, Southampton, Hampshire SO16 6YD, UK; ^3^Faculty of Health Sciences, University of Southampton, Southampton SO17 1BJ, UK

## Abstract

*Purpose*. To trial four-week's physiotherapy targeting chair transfers for people with Parkinson's disease (PwPD) and explore the feasibility of reliance on remote outcome measurement to preserve blinding. *Scope*. We recruited 47 PwPD and randomised 24 to a focused home physiotherapy programme (exercise, movement strategies, and cueing) and 23 to a control group. We evaluated transfers (plus mobility, balance, posture, and quality of life) before and after treatment and at followup (weeks 0, 4, 8, and 12) from video produced by, and questionnaires distributed by, treating physiotherapists. Participants fed back via end-of-study questionnaires. Thirty-five participants (74%) completed the trial. Excluding dropouts, 20% of questionnaire data and 9% of video data were missing or unusable; we had to evaluate balance *in situ*. We noted trends to improvement in transfers, mobility, and balance in the physiotherapy group not noted in the control group. Participant feedback was largely positive and assessor blinding was maintained in every case. *Conclusions*. Intense, focused physiotherapy at home appears acceptable and likely to bring positive change in those who can participate. Remote outcome measurement was successful; questionnaire followup and further training in video production would reduce missing data. We advocate a fully powered trial, designed to minimise dropouts and preserve assessor blinding, to evaluate this intervention.

## 1. Introduction

Chair transfers, a common cause of falls [1, 2], are a key domain of physiotherapy for people with Parkinson's disease (PwPD) [3–5]. While weak lower limbs and inflexible, unstable trunks extend rising time [6–10], exercise shortens sit-to-stand times and PwPD can relearn motor sequences, facilitating movement through cueing [3, 4, 11–14]. 

In their 2007 evidence-based analysis of physical therapy in Parkinson's disease (PD), Keus et al. [3] found supportive evidence for improving the performance of transfers among PwPD in just two studies. The potential to improve transfers among PwPD has been underresearched since Kamsma et al. [11] and Nieuwboer et al. [12] evaluated the use of cognitive movement strategies, the former in a randomised controlled trial (RCT; *n* = 38), the latter in a nonrandomised controlled trial (*n* = 33). Over 12 months, Kamsma et al.'s experimental group (mean age 68 years) practiced a seven-step sequence for safe rising (positioning hands, positioning feet, shifting to the seat edge, repositioning hands, leaning forward, rising into standing, and adopting upright posture), a “logical structure” that offered “maximum opportunity for controlled execution without time constraints.” Participants were found able to learn and demonstrate the strategies and they reported their use in real-life situations, though the effects of training were activity-specific. Nieuwboer et al.'s participants (mean age 66 years) undertook six weeks of home-based physiotherapy aimed at reducing specific difficulties during functional activities including rising which was based on cueing, conscious control of movement, biomechanical compensation, and repetition of movement in differing circumstances. The chair rising strategy taught entailed repositioning “the centre of mass in relation to the base of support to compensate for slow trunk flexion and insufficient horizontal momentum.” Chair transfers improved significantly after treatment, when measured against the Parkinson's Activity Scale (PAS) [13]. More recently, Mak and Hui-Chan [14], recruited 60 PwPD to an RCT comparing rising times after four week's audio-visual cued task-specific sit-to-stand training, four week's conventional mobility and strengthening exercise (for the trunk and lower limbs, followed by sit-to-stand training), and no treatment. Rising times shortened after treatment in both the cued group (mean age 63 years) and the exercise group (mean age 66 years), by 25% and 10%, respectively.

In 2006, the National Institute for Health and Clinical Excellence called for further trials to investigate physiotherapy in PD [15]; however, trials overly focused on measuring cost-effectiveness and quality of life might overlook meaningful changes in performance that are the realistic targets of physiotherapy, potentially reducing the chances of PwPD accessing appropriate therapies. Blinding assessors is “one of the methodological safeguards” that ensures a trial's “internal validity” [16]: inadvertent “unblinding” is an important issue in rehabilitation research. In physiotherapy trials, an assessor's blinding is jeopardized when a participant says or does something that hints at, or confirms, their group allocation: as face-to-face or telephone contact between assessor and participant is highly likely to break blinding, we investigated remote evaluation of video- and questionnaire-based measures by a blinded assessor. 

In a feasibility RCT, we investigated whether focused physiotherapy (cueing, movement strategies, and exercise), increased transfer independence and speed while reducing difficulty and brought secondary changes in gait, balance, posture, and quality of life. Key issues were the programme's potential for a full-sized trial (we did not power this study to test the significance of differences between groups or over time) and the feasibility and acceptability of methods (including the acceptability of participating in research at home). In keeping with recent physiotherapy studies involving PwPD [17, 18], we opted for a home-based trial, firstly to avoid participant travel being a barrier to anyone's participation and secondly, as specialists advocate the delivery of physiotherapy at home [4, 12] where “activities are proving problematic” [19]. 

## 2. Methods

We recruited PwPD from a clinic and support groups within Hampshire who

1. had a working diagnosis of PD, stages I to IV [20], fulfilling the UK PDS Brain Bank diagnostic criteria [21]. The staging allowed us to compare grossly the spectrum of PD in the intervention and control groups:
  - stage I indicating mild unilateral symptoms,
  - stage II, bilateral symptoms without balance impairment,
  - stage III, postural instability but independently mobile,
  - stage IV, severe PD although able to stand and walk with assistance;
2. self-reported chair transfers as
  - being excessively slow *and/or, *
  - requiring much effort, assistance, or repeated attempts *and/or, *
  - associated with a previous fall;
3. scored at least 8/12 on The Middlesex Elderly Assessment of Mental State [22], a gross screen for cognitive impairment that we have used in previous studies to identify anyone with PD at risk of being unable to give fully informed consent [2, 23] and that is one of the most commonly used assessments by occupational therapists of mental state in PwPD [24];
4. were willing and able to undertake all aspects of the intervention;
5. were willing and able to complete the outcome measures (albeit with help from another person in completing questionnaires, if handwriting was problematic).

Southampton and South West Hampshire Ethics Committee approved the project and all participants gave written informed consent to all aspects of the study, including specifically video recording. After responding to an initial invitation to take part, interested parties were visited at home by one of three treating physiotherapists. Having given their consent, a therapist completed the participant's baseline (week 0) assessment, after which the participant was randomised to either the intervention group (receiving physiotherapy) or the control group (receiving none) through concealed allocation.

### 2.1. Intervention

The physiotherapy group undertook a four-week-long, evidence-based [3, 11–14], home physiotherapy programme focused on chair transfers, comprising (1) supervised exercise (to enhance hip and knee extensor strength and trunk stability and flexibility), (2) teaching and learning movement strategies for safer and easier standing and sitting, and (3) verbal cueing. The intervention was not novel in content but in its intense, focused delivery. Respecting individuality and professionalism, the protocol dictated only that the physiotherapists provided no more than 12 hours of input focused only on chair transfers (a maximum of one hour, three times per week for four weeks) including only portable equipment (like ankle weights); the therapists decided if, when, and how intensively to use exercise, strategies, and/or cueing based on each participant's assessment and their clinical experience. The primary objective was to improve the ability to transfer, in terms of independence, speed (timed sit-to-stand), and/or difficulty. Improvements in mobility, balance, posture, or quality of life would be secondary benefits.

### 2.2. Assessment

We assessed participants four times, at the same time of day and point during an “on” phase, before and after intervention (weeks 0 and 4) and during followup (weeks 8 and 12). The physiotherapists' video-recorded five physical performance tests for the blinded assessor to evaluate: PAS; sit-to-stand [14]; Standing Start 180 Degree Turn Test (SS-180) [25]; functional reach (FR) [26]; the Unified PD Rating Scale Posture Item [27]. All five tests have been used previously with PwPD (and three were developed specifically for PwPD), and we followed the published protocols without modification. A single camera, stopwatch, and metre-rule were used across the study but, as a home-based study, the chairs that participant's rose from during the PAS varied (but each person used their same chair at every assessment point).

The physiotherapists' also left the participants two questionnaires to complete at home and return in a stamped addressed envelope: PD Self-Assessed Disability Scale (SAS) [28] and 15D instrument of health-related quality of life (HR-QOL) [29]. On SAS, participants indicated on a five-point scale how much effort 25 everyday tasks took: best score 25, worst score 125. On HR-QOL, they indicated on a five-point scale the state of 15 aspects of health: best score 15, worst score 75. 

The physiotherapists transferred edited clips of each performance (but nothing superfluous) onto DVD for one independent assessor (blind to group allocation) to evaluate, alongside returned questionnaires and feedback. Independence and effort demonstrated during the PAS Chair Transfer section was rated from zero to eight (worst to best score). Sit-to-stand was stopwatch-timed (during the PAS) from the point when a participant started to move until they attained stable standing. The SS-180 mean turn time was calculated from two turns (one in each direction) to walk towards a target. FR was the mean of three maximal forward reaches in standing. Posture was rated as the degree of stoop noted when participant's stood for the FR, rated zero to four (best to worst score). Sound was turned off during evaluation to prevent inadvertent unblinding but restored afterwards on a sample to quality check that the therapists had used standard instructions during data collection. During piloting, we found it unacceptable to evaluate FR from video, as the numbers on the metre-rule were (1) too small to read in the wide image necessary to rate posture and quality-check test conduct and (2) obscured by the reaching arm. So during the RCT physiotherapists rated FR *in situ*. 

In their final week of involvement, we gave participants an anonymous feedback form (including space to comment) posing the following questions about the acceptability of home-based research, the randomisation, intervention, and assessments.

Did you find it difficult to fit taking part in the study into your routine?
Was it difficult to find enough space for video recording at home?
How do you feel about not being in the group that had physiotherapy?
Did you find physiotherapy helpful (and why)?
Do you think you had too little, enough, or too much physiotherapy (and why)?
Did you feel comfortable with video recording?
Were the tests quick enough to complete?
Were the questionnaires boring *or* difficult to complete *or* difficult to post back?

## 3. Results

We recruited 47 PwPD (median age 74 years; median years since diagnosis seven), including 45 who found transfers excessively slow, 39 who found transfers an excessive effort, and 17 who had fallen transferring; 13 participants (28%) reported all three indicators. Participant median Hoehn and Yahr stage was III and half the participants had fallen repeatedly in the previous year. The control group (*n* = 23) and treatment group (*n* = 24) were similar in characteristics and baseline performances (Table 1). 

Thirty-five participants (74%) completed: four (9%) dropped out by week 4, five (11%) by week 8, and 12 (26%) by week 12. Of the final 12 drop-outs, eight (67%) were from the treatment group, through illness. Dropouts were representative of the whole sample age (median 73 years) and years since diagnosis (median 8) but 11/12 (92%) were at Hoehn and Yahr stage III or IV (in comparison with 79% of the whole sample). 

Over the intervention period (weeks 0 to 4), as outlined in Table 2, the physiotherapy group median PAS score tended to improve (from 4 to 6) while that of the controls tended to worsen (from 6 to 4). The tendency to improvement in the physiotherapy group continued throughout followup (median score reaching 7 by week 12) while the control median returned to baseline. Median sit-to-stand times tended to shorten from weeks 0 to 4 (by 14%, from 2.2 s to 1.9 s, in both groups) and to continue shortening to week 12 (reaching 1.5 s in the physiotherapy group and 1.7 s in the controls, reductions of 32% and 23% from baseline, resp.). The physiotherapy group median SAS score tended to improve slightly by week 4 (by one point, from 50 to 49) while that of the controls tended to worsen (by 7 points, from 52 to 59); median scores tended to worsen in both groups by week 12 (the physiotherapy group deteriorating from baseline by 2 points, the controls by 12 points). 

Over the intervention period, the physiotherapy group median SS-180 time tended to shorten (by 17%, from 5.3 s to 4.4 s) while that of the controls tended to lengthen (by 5%, from 3.7 s to 3.9 s). Throughout followup, the tendency to improvement in the physiotherapy group continued (median reaching 3.8 s by week 12, a reduction of 28% from baseline) while controls turn time remained longer than at baseline. The physiotherapy group median FR tended to improve slightly by week 4 (15%, from 19.2 cm to 22.0 cm) while that of the controls changed minimally (0.5%, from 20.9 cm to 21.0 cm); the tendency to improvement in the physiotherapy group continued (median reaching 25.5 cm by week 12, a 33% increase from baseline) while the control's worsened (to median 19.7 cm, a 6% decrease from baseline). We detected little change in posture or quality of life in either group. On the UPDRS posture item, both groups were rated a median 1 (IQR 1-2) at every assessment point. The median HR-QOL score of both groups worsened by one point (from 29 to 30) by week 4; at week 12 the physiotherapy group median had returned to baseline while that of the control group was two points worse.

Thirty-nine participants (83%) returned anonymous feedback forms at the end of the study, 20 from the physiotherapy group and 19 controls. Few participants found difficulty fitting in the study or finding space for the video-based assessments (3/35 in each case). Of 17 controls, seven expressed disappointment about not being in the treatment group (*“I would have liked to see if it could help my condition”*), while ten were ambivalent, for example, *“someone had to be in this group and I was one of them.”* Two of the treatment group (10%) found the intervention unhelpful but 18 (90%) found it helpful, reporting they had

1. learned new skills or exercises (*“Despite long-term Parkinson's I still learned new ways”*),
2. found movement or exercise easier (*“Easy to follow instruction, made easier by being in my home; I put them into practice during my daily round; they have become part of my routine”*),
3. gained useful advice and support from a good therapist (*“Guidance managing my disability”*).

Eight (40%) felt they had insufficient therapy time, as one hour was an inadequate representation (*“She never saw me at my worst”*) or they needed encouragement (*“Physiotherapy prompts me into being regular with my own efforts”*) and “the more the better” (*“It works well at the time and more would have been an advantage”*). But twelve (60%) suggested it fitted their routine (*“I was not overburdened”*), they were tired afterwards (*“Quite tired by the end”*), and more might have been repetitive (*“I did not feel bored or disinterested”*). Most felt comfortable being video-recorded (35/36) and found assessment acceptably quick (33/35). Few found the questionnaires difficult to complete (2/35) or return (1/39) or found them boring (2/36). Participants wrote few comments; although overwhelmingly positive, one participant felt *“filming was unrepresentative; she never saw me totally unable to move or having to crawl on the floor.”*

Reliance on video and questionnaires preserved assessor blinding fully: the assessor learned no-one's group allocation as there were no distinguishing features in 600-plus silent video clips. 

Of a potential 1316 measurements (seven outcomes measured for 47 participants four times), 131 (10%) were missing as participants had dropped-out (Table 3); of the remaining measurements, 185/1185 (16%) were missing or unusable. FR was the only test someone declined to attempt, and a documentation error (later rectified) invalidated 14% of potential FR data. Of the tests evaluated from video, the SS-180 had most missing/unusable data, 15% after excluding dropouts: records were discounted if the protocol had been followed incorrectly (e.g., the participant turned in the same direction on both trials), if editing invalidated the clip (e.g., ending before the turn was complete) or if the recording was inadequately lit. Timed sit-to-stand, PAS, and posture score had percentages of unusable data below 10%, after excluding dropouts. 

## 4. Discussion

This study revealed a trend to improved transfers, mobility, and balance among PwPD after physiotherapy. It would be feasible to deliver this focused programme quickly and easily in the home. Over a quarter of participants had multiple difficulties with transfer speed, effort, and stability, all of which physiotherapists can address, yet Keus et al. [30] found transfers to be a physiotherapy priority in just 14% of cases, behind gait (74%), posture (49%), and balance (37%). While controls deteriorated, our intervention group tended to continue to improve over followup (sit-to-stand time decreased 14% by week 4 and 32% by week 12), which suggests continual refinement of newly learned strategies. As the need is evident and intervention possible, as suggested by these results and others [1–14], transfer training (preferably at home, where people can deploy strategies learned) should be integral to physiotherapy for PwPD. Illness, fatigue, and a lack of perceived benefit are commonly reasons why PwPD discontinue exercise regimes [31]. Illness among our treatment group accounted for most dropouts (as was the case when Nieuwboer et al. [12] lost 15% of their recruits to a home-based physiotherapy trial over 12 weeks), and some participants reported extreme fatigue after physiotherapy: this intervention warrants selective application. 

The importance of physiotherapists' expertise and relationships with patients in the quality and outcome of treatment is well recognised [30, 32]. From feedback, our sample appeared pro-physiotherapy (they learned new skills and ways of managing their condition) and pro-research (they understood their role within the study). Although several controls were disappointed, none dropped out following randomisation. Experienced clinicians in physiotherapy trials enhance the participant experience and data collection.

Among our video-based evaluations, drop-outs accounted for two-thirds of the missing data; technical difficulties with video production errors (such as filming in too dark a setting or in too confined or cluttered a space or editing the clip prepared for the blinded assessor too harshly) and protocol/documentation errors (such as having the person performing the SS-180 turn twice in the same rather than the opposite direction or recording the FR as habitually done during clinical practice rather than in the standard way used in the study) accounted for the rest. The layout, light levels, and contents of an individual's home are their choice and researchers cannot expect the ideal conditions for data collection that they might expect in a purpose-built movement laboratory; although they can prepare the area used for video-recording to a certain extent, it is not reasonable to impose whole-scale modifications. It can be difficult to know at the time of recording that a clip will be too poorly illuminated for the assessor when they see it later. Similarly, a participant may move out of the camera's scope in an unexpected way that only comes to light when editing the clip. The more complex the test, the more likely it is that a proportion will be obscured or lost. On balance, we believe that losing a proportion of data recorded in the home is outweighed by the benefits of the inclusivity that home-based research offers to the people who wish to participate. Specific training and monitoring of therapists would improve these aspects of data collection. This feasibility study revealed that while experienced clinicians make good researchers, they are unlikely to have been trained in all the necessary research skills beforehand and they are also likely to have developed ways of conducting and recording tests in their professional practice that differ from the study protocols. Again, on balance, we believe that the benefit of employing experienced clinicians outweighs the costs involved in employing and training them in research skills which may be entirely new.

FR was the only test declined for fear of falling (by one individual on three occasions) and was the only assessment not feasible in the home, in this study. After dropouts, the major causes of missing/unusable questionnaire data were unreturned questionnaires (13% were missing) and incomplete questionnaires (omitted answers invalidated 9% of otherwise complete HR-QOL questionnaires). Feedback did not indicate problems but prompts or collection (both with cost and ethical implications) would have increased returns. Haapaniemi et al. [29], who received 15% of their HR-QOL questionnaires incomplete, used regression analysis to predict missing data, an option we would advocate. 

Our assessment battery, which took approximately 20 minutes to complete in the home, measured the difficulty, slowness, and dependence associated with transfers. Speed improved by week 4 in both our groups, associated with an improvement in PAS score in the treatment group and deterioration among the controls. Others have demonstrated changes in transfer strategy after training while speed remains unchanged [33] and have stressed that function and stability are more important than speed [4]. We could recommend both the PAS score and sit-to-stand time as primary outcome measures in future, as the demand on participants is low and evaluation from video was associated with relatively little missing data. In light of the numbers of unreturned and incomplete questionnaires, and its breadth, we would not advocate the SAS in a similar way. 

While it may be impossible to blind a trial participant as to whether they have actively taken part in a physiotherapy intervention [34], it should be possible to keep the assessor blind. Even when it is possible to blind the assessor, this is not always done or reported. In the present study, using silent video and questionnaires preserved total assessor blinding and was associated with other advantages over face-to-face or telephone contact. Working without distraction from edited clips reduced the time (and money) spent travelling to, and engaging with, participants by the experienced assessor. Video facilitates reliability testing, team review, illustration of findings, and quality control. Participants found the assessments acceptable, and (with the exception of FR) the assessments were feasible using standard equipment. Evaluation of video by a blinded assessor has been used successfully in fields such as gastric surgery [35] and pain control [36]; here we have demonstrated its feasibility in movement analysis. Video should only be used with explicit consent ensuring precautions are taken to (a) avoid recording anyone else's image or conversation and (b) maintain the subject's safety [37]: a tripod-mounted, battery-powered camera is safer than one which occupies the recorder's hands and presents a trip hazard. 

We make the following recommendations for a future RCT. Employ experienced clinicians, teach them the required research skills, and monitor fidelity to the recording protocol throughout the trial. When calculating a sample size, consider that our 19 controls scored a mean 5.11 (1.97) on the PAS Chair Transfer at baseline, which *decreased *a mean 0.32 (1.29) to a mean 4.79 (1.47) at week 4; the intervention group (*n* = 20) scored a mean 4.55 (1.39) at baseline, which *increased* a mean 0.90 (1.80) to a mean 5.45 (1.61) at week 4. Over-recruit by 25% to compensate for dropouts. Offer controls some intervention, in light of the multiple transfer difficulties of PwPD and our controls' feedback. Use more sensitive measures of posture (such as tragus-to-wall distance) and quality of life than we trialled. As specifically *supervised* exercise was a component of the intervention in this trial, we did not ask participants to report any *unsupervised* exercise they may have undertaken; we suggest that researcher's consider recording the latter in a full RCT. While the performances evaluated in this study were analysed by one blinded assessor, they were recorded by the unblinded trial physiotherapists: while the recordings allowed us to quality check test conduct, employing an independent recorder to collect and prepare clips for the assessor might reduce potential bias even further.

## 5. Conclusion

If the measures are suitable, an intense, focused physiotherapy programme delivered at home by experienced physiotherapists is likely to bring about positive change in those who can participate. We recommend a fully powered trial (over-recruiting to offset dropouts and offering the controls an incentive to remain) using remote outcome measurement (especially silent video assessment), as far as possible, to preserve blinding: missing/unusable data can be reduced by comprehensive training in video recording/editing and following up/collecting questionnaires or replacing missing answers using statistical methods. The key implication of this feasibility trial is that a sample of PwPD older than in the earlier studies discussed, appeared to derive benefit from a programme that was shorter than most of the earlier studies and one that practicing clinicians could roll out without additional training or equipment.

## Figures and Tables

**Table 1:** Participant characteristics at baseline (*n* = 47).

Variable	Value	Control group (*n* = 23)	Treatment group (*n* = 24)	All (*n* = 47)
Age (years)	Median (IQR), range	74 (70–78), 58–86	75 (69–77), 64–82	74 (69–77), 58–86
Gender	Men (*n*)	18 (78%)	17 (71%)	35 (74%)
Years since diagnosis	Median (IQR), range	7 (4–12), 1–19	8 (4–11), 1–30	7 (4–12), 1–30
Hoehn and yahr (grade)	I	1	0	1 (2%)
	II	5	4	9 (19%)
	III	10	12	22 (47%)
	IV	7	8	15 (32%)
UPDRS	Median (IQR), range	30 (18–45), 9–52	26 (21–38), 10–60	28 (20–41), 9–60
12-month fall history	No falls (*n*)	7	4	11 (23%)
	Single fall (*n*)	6	6	12 (26%)
	Repeated falls (*n*)	10	14	24 (51%)

Indication for physiotherapy				
Transfers excessively slowly	*n* (%)	23 (100)	22 (92)	45 (96)
Transfers a considerable effort	*n* (%)	20 (87)	19 (79)	39 (83)
History of falls transferring	*n* (%)	10 (43)	7 (29)	17 (36)

Primary outcome measures				
PAS chair transfer score	Median (IQR), range	5 (4–6), 0–8	4 (4–6), 2–8	4 (4–6), 0–8
Sit-to-stand time (s)	Median (IQR), range	2.2 (1.6–3.7), 0.8–11.1	2.1 (1.5–3.2), 0.8–7.2	2.2 (1.5–3.2), 0.8–11.1
SAS score	Median (IQR), range	54 (41–70), 37–104	50 (43–63), 36–90	51 (41–65), 36–104

Secondary outcome measures				
SS-180 turn time (s)	Median (IQR), range	3.8 (3.4–6.8), 1.8–45.6	5.5 (3.8–8.4), 2.2–43.5	5.3 (3.5–7.4), 1.8–45.6
FR (cm)	Median (IQR), range	21 (17–25), 10–33	18 (16–21), 9–32	20 (16–23), 9–33
UPDRS posture score	Median (IQR), range	1 (1-2), 0–4	1 (1-2), 0–3	1 (1-2), 0–4
HR-QOL score	Median (IQR), range	30 (26–32), 21–44	30 (28–37), 16–47	30 (27–35), 16–47

IQR = interquartile range; range = minimum to maximum.

**Table 2:** Changes in outcomes (weeks 0–12, by group) in participants who completed a measure on at least three occasions; values presented are medians (interquartile range).

Primary outcomes	Group	Week 0	Week 4	Week 8	Week 12
PAS chair transfer (score)	Control (*n* = 18)	6 (4–7)	4 (4–6)	6 (4–7)	6 (3–7)
	Physiotherapy (*n* = 19)	4 (4–6)	6 (4–7)	7 (5–8)	7 (4–8)
Sit-to-stand time (s)	Control (*n* = 20)	2.2 (1.4–3.2)	1.9 (1.3–2.8)	2.0 (1.4–2.2)	1.7 (1.3–2.2)
	Physiotherapy (*n* = 18)	2.2 (1.6–3.1)	1.9 (1.4–2.0)	1.7 (1.0–2.4)	1.5 (1.2–2.0)
SAS (score)	Control (*n* = 18)	52 (40–64)	59 (45–71)	60 (48–66)	64 (50–77)
	Physiotherapy (*n* = 16)	50 (43–59)	49 (43–67)	58 (50–66)	52 (43–60)

Secondary outcomes					
SS-180 turn time (s)	Control (*n* = 16)	3.7 (3.1–6.8)	3.9 (3.1–7.0)	4.1 (2.7–9.6)	3.9 (2.8–5.9)
	Physiotherapy (*n* = 16)	5.3 (3.9–6.7)	4.4 (3.4–6.4)	3.7 (3.4–4.8)	3.8 (3.1–6.1)
FR (cm)	Control (*n* = 16)	20.9 (15.7–25.2)	21.0 (15.0–24.3)	21.7 (13.6–25.8)	19.7 (17.4–27.7)
	Physiotherapy (*n* = 13)	19.2 (17.5–21.9)	22.0 (20.0–25.0)	22.8 (20.3–25.8)	25.5 (19.6–30.2)
UPDRS posture (score)	Control (*n* = 19)	1 (1-2)	1 (1-2)	1 (1-2)	1 (1-2)
	Physiotherapy (*n* = 19)	1 (1-2)	1 (1-2)	1 (1-2)	1 (1-2)
HR-QOL score	Control (*n* = 14)	29 (26–31)	30 (25–33)	29 (25–33)	31 (24–34)
	Physiotherapy (*n* = 14)	29 (26–36)	30 (28–36)	32 (28–38)	29 (27–34)

**Table 3:** Reasons for missing and unusable data, by measure type.

Measure type	Number of potential measurements	Number of data missing		Number of data unusable
		Participant drop-out	Test declined, omitted, or not recorded or questionnaire not returned	Error in testing or recording, poor-quality recording, or questionnaire incomplete

*In situ—real time*				
FR	188	19 (10%)	18 (10%)	27 (14%)

*Video based*				
PAS	188	19 (10%)	9 (5%)	4 (2%)
Transfer time	188	19 (10%)	9 (5%)	2 (1%)
SS-180 time	188	19 (10%)	10 (5%)	18 (10%)
Posture	188	19 (10%)	9 (5%)	5 (3%)

*Postal questionnaires*				
SAS	188	18 (10%)	24 (13%)	9 (5%)
HR-QOL	188	18 (10%)	25 (13%)	16 (9%)

Total	**1316**	**131 (10%)**	**104 (8%)**	**81 (6%)**
